# Characterizing the Role of Monocytes in T Cell Cancer Immunotherapy Using a 3D Microfluidic Model

**DOI:** 10.3389/fimmu.2018.00416

**Published:** 2018-03-06

**Authors:** Sharon Wei Ling Lee, Giulia Adriani, Erica Ceccarello, Andrea Pavesi, Anthony Tanoto Tan, Antonio Bertoletti, Roger Dale Kamm, Siew Cheng Wong

**Affiliations:** ^1^BioSystems and Micromechanics IRG, Singapore-MIT Alliance for Research and Technology, Singapore, Singapore; ^2^Department of Microbiology and Immunology, Yong Loo Lin School of Medicine, National University of Singapore, Singapore, Singapore; ^3^Singapore Immunology Network (SIgN), Biomedical Sciences Institute, Agency for Science, Technology, and Research, Singapore, Singapore; ^4^Institute of Molecular and Cell Biology, Agency for Science, Technology, and Research, Singapore, Singapore; ^5^Programme of Emerging Infectious Diseases, Duke-NUS Medical School, Singapore, Singapore; ^6^Department of Biological Engineering, Massachusetts Institute of Technology, Cambridge, MA, United States

**Keywords:** microfluidics, monocytes, T cell receptor-redirected T cells, immunotherapy, immune checkpoint, PD-L1, tumor microenvironment

## Abstract

In the hepatitis B virus (HBV)-related hepatocellular carcinoma tumor microenvironment (TME), monocytes reportedly impede natural T cell functions *via* PD-L1/PD-1 signaling. However, it remains unclear if T cell receptor-redirected T cells (TCR T cells) are similarly inhibited. Hence, we developed a 3D intrahepatic TME microfluidic model to investigate the immunosuppressive potential of monocytes toward HBV-specific TCR T cells and the role of PD-L1/PD-1 signaling. Interestingly, in our 3D static microfluidic model, we observed that monocytes suppressed only retrovirally transduced (Tdx) TCR T cell cytotoxicity toward cancer cells *via* PD-L1/PD-1, while mRNA electroporated (EP) TCR T cell cytotoxicity was not affected by the presence of monocytes. Importantly, when co-cultured in 2D, both Tdx and EP TCR T cell cytotoxicity toward cancer cells were not suppressed by monocytes, suggesting our 3D model as a superior tool compared to standard 2D assays for predicting TCR T cell efficacy in a preclinical setting, which can thus be used to improve current immunotherapy strategies.

## Introduction

Adoptive cell therapy (ACT) consists of the administration of lymphocytes to cancer patients to mediate an anticancer effector response. Currently, the capability to genetically engineer lymphocytes to express T cell receptors or chimeric antigen receptors has further advanced ACT for cancer treatment (1–6). T cells engineered to express TCRs specific for viral antigens could represent a relevant therapeutic tool against tumor cells expressing viral antigens. Qasim et al. demonstrated the clinical potential of hepatitis B virus (HBV)-specific T cell receptor-redirected T cells (TCR T cells) for the compassionate treatment of a patient with HBV-related hepatocellular carcinoma (HBV-HCC) metastatic disease. The adoptive transfer of the HBV-specific TCR T cells resulted in a 90% reduction of circulating HBsAg after 4 weeks of treatment with no collateral side effects (4). Robbins et al. also demonstrated the effectiveness of TCR T cells in the treatment of melanoma and synovial sarcoma (7). Previous studies, however, have shown that different cell engineering protocols result in TCR T cells that can differ significantly in both antitumor efficacy and their interactions with the tumor microenvironment (TME) (8–10). In particular, studies have demonstrated that HBV-specific TCR T cells are phenotypically different when produced by either retroviral transduction or mRNA electroporation, with each TCR T cell type also carrying distinct advantages for therapy (11–14).

In cancer, TME-associated factors that are known to inhibit effector T cell functions include immunosuppressive stromal cells and the expression of programmed death ligand-1/programmed death-1 (PD-L1/PD-1) on both cancer cells and myeloid-derived cells (15, 16). A poorer disease prognosis has been observed for patients with either higher PD-L1 expression levels or a higher proportion of PD-1^+^ CD8^+^ T cells in the TME (17–21). PD-L1 has been described as a “molecular shield” that protects tumor cells from T cell-mediated eradication (22). Moreover, interfering with the PD-L1/PD-1 axis has been shown to augment the cytotoxic activity of CD8^+^ T cells (19, 20, 22, 23). Recently, in fact, strategies utilizing antibodies targeting the PD-L1/PD-1 axis have been approved for use in patients after encouraging clinical trials (24–27). However, such findings are based on physiological tumor-infiltrating leukocytes (TILs), while the contribution of PD-L1-based signaling on TCR T cell functions remains to be investigated.

Monocytes/macrophages constitute a major component of the tumor stroma and are known to importantly modulate effector T cell activity *via* PD-L1 (19, 28, 29). Notably, in response to TME-specific signals, monocytes can acquire unique phenotypes and functions to become tumor-associated macrophages (30–32). Studies concur that monocytes are only capable of a weak and short-lived antitumor response and, instead, predominantly display protumor and immunosuppressive functions (33–35). However, the inherent plasticity of monocytes implies that these cells could elicit a heterogeneous response.

Murine models are widely used in research to study the interactions between TILs and the TME (36–39). While such models provide a useful tool in elucidating the mechanisms underlying cancer pathology and immune evasion in a highly physiological manner, it is not feasible to use them in a clinical setting to rapidly evaluate the efficiency of therapeutic T cells. This is because murine models are high in cost, challenging to handle, require several months to develop, and may still not fully recapitulate the complexity of the human system. Particularly, for the field of HBV-HCC, no reliable and physiologically relevant murine model currently exists (39, 40).

Alternatively, there are 2D or 3D *in vitro* tumor models. A recent review (41) showcased in detail numerous 3D tumor models including spheroids or organoids, microfluidic culture systems, and filter-supported or paper-supported multilayer cultures (e.g., Transwell) (41). Microfluidic platforms mimic important physiological cues through the architectural support of a 3D extracellular matrix-like hydrogel. Such platforms also have distinct advantages over conventional 3D cultures in well or Transwell configuration such as (i) a reduction of reagents and biological components with relative cost savings, (ii) a better accessibility for live imaging with standard microscopes, (iii) the possibility to create chemical gradients, and (iv) increased cellular and architectural complexity such as the co-culture of tumor cells with endothelial, stromal, and immune cells (42–49). For our purpose of studying cellular interaction, it is also fundamental to eliminate *in vitro* artifacts such as the gravity-mediated interactions between cells that occur in conventional 3D Petri dish or Transwell migration assays. Therefore, considering the general limitations derived from the use of experimental models, a 3D microfluidic TME model not only bridges the gap between classical *in vitro* systems and current *in vivo* models but also could serve as a rapid and efficacious tool in the preclinical evaluation of TCR T cells for personalized treatment.

**Figure 1:**
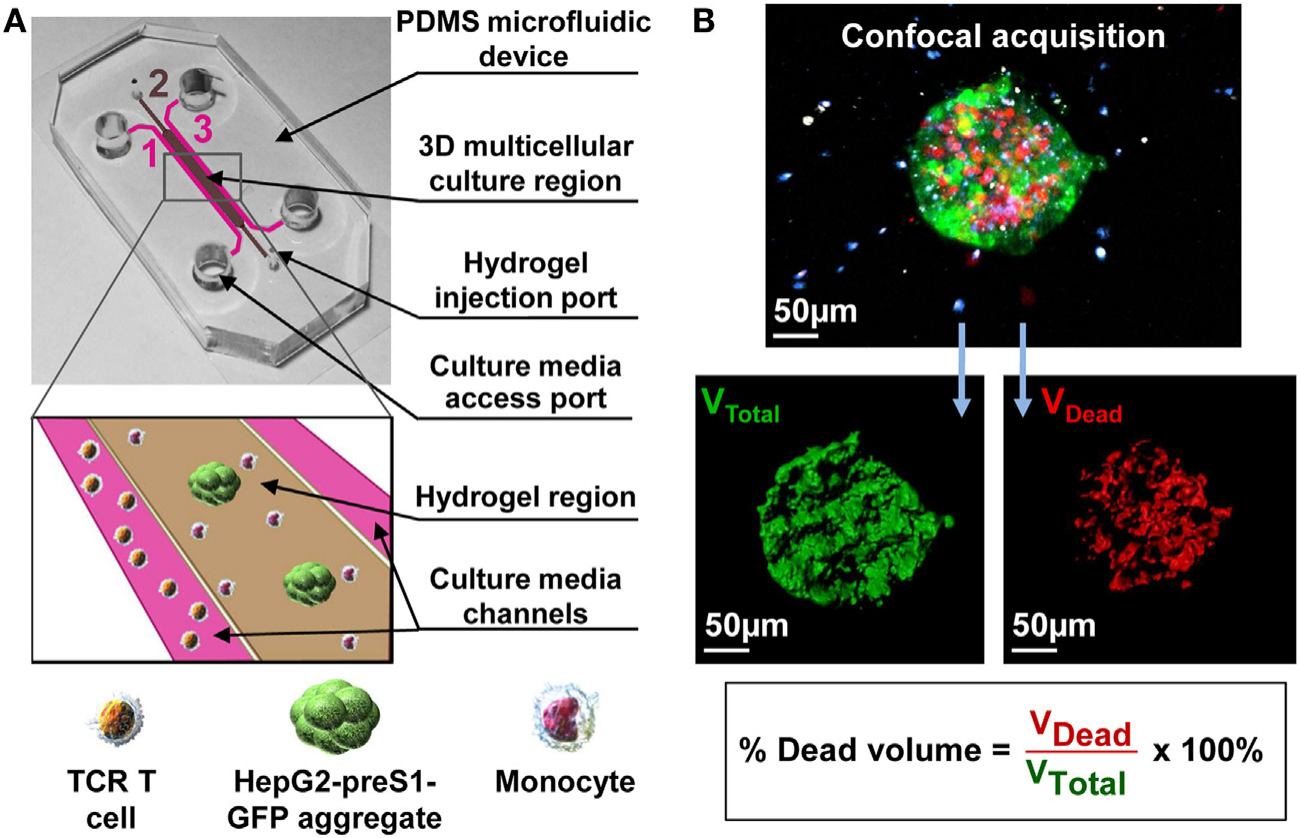
**(A)** A 3D multicellular tumor microenvironment microfluidic model consisting of a middle hydrogel channel (2) flanked by two media channels (1, 3) for the mechanistic study of the effect of monocytes on T cell receptor-redirected T cell (TCR T cell) killing of tumor cell aggregates. Human monocytes were inserted together with target HepG2-preS1-GFP cell aggregates in collagen gel in the central hydrogel region (2), while hepatitis B virus (HBV)-specific TCR T cells were added into one fluidic channel (1) to mimic the intrahepatic carcinoma environment. **(B)** Representative confocal image of a target cell aggregate (in green) surrounded by monocytes (in blue) and HBV-specific TCR T cells (in white), in which the presence of dead target cells is DRAQ7^+^ (in red). Target cell death is quantified as shown based on the DRAQ7^+^ volumetric portion in the total volume of each GFP-labeled aggregate.

In this study, a 3D microfluidic platform to recapitulate the HBV-HCC environment is developed to investigate the impact of human primary monocytes on the killing efficacy of HBV-specific TCR T cells (Figure 1A). More specifically, this study explores the effect of monocytes on the killing efficacy of HBV-specific TCR T cells that are produced by different methods and investigates the contribution of PD-L1/PD-1 expression toward the interplay between these cells. We show that our 3D microfluidic model provides a setting with an improved physiological edge over standard 2D systems to investigate tumor-immune cell behavior and is extremely useful for unraveling the impact of certain biological pathways on monocyte–TCR T cell interactions.

## Materials and Methods

### Cell Culture

A human HCC cell line, HepG2, was transduced with a construct containing the preS1 portion of the genotype D HBV envelope protein gene covalently linked to GFP (HepG2-preS1-GFP) using the Lenti-X™ HTX packaging system (Clontech, ST0282) according to the manufacturer’s instructions. HepG2-preS1-GFP cells were cultured in R10 culture medium: RPMI 1640 (ThermoFisher Scientific, 21870076) supplemented with 10% heat inactivated fetal bovine serum (FBS; ThermoFisher Scientific 10082147), 20 mM Hepes (ThermoFisher Scientific, 15630080), 1 mM sodium pyruvate (ThermoFisher Scientific, 11360070), 100 IU/mL penicillin (ThermoFisher Scientific, 15070063), 100 µg/mL streptomycin (Sigma-Aldrich, S9137), MeM amino acids (ThermoFisher Scientific, 11130051), Glutamax (ThermoFisher Scientific, 35050061), MeM non-essential amino acids (ThermoFisher Scientific, 11140050), 5 µg/mL Plasmocin (InvivoGen, ant-mpt), and 5 µg/mL of puromycin (Takara, 631305) to select for transgene expressing target cells (11). HepG2-preS1-GFP was used in experiments at passage 9–11. HepG2.2.15 cell line supports the full HBV replication and was cultured in D10 culture medium: DMEM (ThermoFisher Scientific, MA11960044) supplemented with 10% heat inactivated FBS, 1 mM sodium pyruvate, 100 IU/mL penicillin, 100 µg/mL streptomycin, MeM non-essential amino acids, 200 µg/mL Geneticin reagent (ThermoFisher Scientific, 10131035) to select for transgene expressing target cells. Both the cell lines were maintained in a humidified CO_2_ incubator at 37°C and 5% CO_2_ (11, 38).

Human peripheral blood mononuclear cells (PBMCs) were isolated from whole blood of healthy donors by Ficoll-Paque (GE Healthcare, 17-1440-02) density gradient centrifugation following the informed consent of donors in accordance with the Declaration of Helsinki. Total monocytes either autologous or allogenic were isolated from PBMCs using the Pan-Monocyte Isolation Kit (Miltenyi Biotec, 130-096-537). Consistently, pure (92 ± 0.5%) and viable (98.4 ± 1%) monocytes were obtained. Alternatively, human PBMCs were used to produce HBV-specific TCR T cells or expand bulk transduced T cells as described in Section “Isolation, Transduction, and Expansion of TCR-Redirected T Cells.” All blood samples and procedures used in this study have been approved by the National University of Singapore Institutional Review Board. All experiments were carried out in accordance with the approved guidelines and regulations.

### Isolation, Transduction, and Expansion of TCR-Redirected T Cells

Retrovirally transduced T cells were produced and expanded as previously described (11). Briefly, after isolation, PBMCs were stimulated with 600 IU/mL of recombinant human interleukin-2 (rhIL-2; Miltenyi Biotec, 130-097-745) and 50 ng/mL of anti-CD3 (eBioscience, 16-0037-81) in AIM-V (ThermoFisher Scientific, 12055091), 2% (v/v) human AB serum (Sigma-Aldrich, H6914) for 48 h, before transduction with a MP-71 vector containing the Vα34 and Vβ3 chains of the HBs183-91-specific (Ts) or the HBcore18-27-specific (Tc) TCR sequences. Bulk transduced T cells were expanded and restimulated using 0.5–1 × 10^6^ TCR-transduced T cells, 2 × 10^5^ irradiated (2,500 rads) T2 cells pulsed with 1 µg/mL of either HBs183-91 peptide (FLLTRILTI) or HBcore18-27 (FLPSDFFPSV) (Genscript, custom synthesized peptides) and 1.8 × 10^6^ irradiated PBMCs as feeders. Cells were cultured for about 2 weeks in AIM-V, 2% human AB serum supplemented with 100 IU/mL of rhIL-2, 10 ng/mL of rhIL-7 (Miltenyi Biotec, 130-095-362), and 10 ng/mL of rhIL-15 (Miltenyi Biotec, 130-095-764). In some experiments, CD8^+^ T cells were enriched through negative selection of transduced T cells using CD4 microbeads (Miltenyi Biotec, 130-045-101) following the manufacturer’s instructions.

### Production of s183-191 TCR mRNA and Electroporation

T cell receptor mRNA was derived, cloned, propagated, and concentrated as previously described (12). 5–10 × 10^6^ PBMCs were activated for 8 days with 600 IU/mL of rhIL-2 and 50 ng/mL of anti-CD3 in AIM-V, 2% human AB serum, and rhIL-2 was increased to 1,000 IU/mL 1 day before electroporation. During electroporation, 10 × 10^6^ cells were suspended in 200 µL of BTXPRESS Low Conductivity Medium T4 (Harvard Bioscience Inc., 47-0003), and TCR mRNA was added at 100 µg/mL. The mixture was placed in a certified cuvette and electroporated using a customized program of the AgilePulse Waveform Electroporation System (Harvard Bioscience Inc., 47-0201N). After electroporation, cells were resuspended in AIM-V, 20% human AB serum supplemented with 100 IU/mL of rhIL-2 and cultured at 37°C and 5% CO_2_ for 24 h until analysis. T cells expressing a TCR recognizing the HBcore18-27 peptide were additionally prepared to be used as a control as previously described (12).

### Flow Cytometry

Antibodies for cell surface staining were obtained from BD Biosciences (anti-human CD8 PE-CF594 562282, CD8 PE-Cy7 557746), Biolegend (anti-human PD-L1 BV711 329722, PD-1 BV421 329920), and eBioscience (anti-human CD14 eFluor450 48-0149, PD-1 APC 17-2799). HBV-specific TCR T cells were analyzed for TCR expression using specific HLA-0201 Env183-91 (FLLTRILTI) pentameric complexes (Proimmune, 027) or HLA-0201 core18-27 (FLPSDFFPSV) dextrameric complexes (Immudex, WB3289).

Before flow cytometry analysis, HepG2-preS1-GFP target cells, monocytes, and HBV-specific TCR T cells were co-cultured in 2D wells for 24 h in AIM-V, 2% human AB serum supplemented with 100 IU/mL of rhIL-2. To stain for PD-L1 or PD-1, cells were collected using a trypsin-free approach. After collecting the supernatant, cells were incubated with PBS/EDTA (PBS; 2 mM EDTA; Axil Scientific, BUF-1052) (7–10 min, 37°C, 5% CO_2_), before rinsing with MACS buffer (PBS, 0.5% BSA, 2 mM EDTA). Cells were stained with live/dead stain (Life Technologies, L34957) diluted in PBS 1:500 (15 min, room temperature) and rinsed twice in PBS before staining for surface markers. Surface staining was performed in FACS buffer [PBS, 2 mM EDTA, 5% FBS, 5% human serum, 0.1% sodium azide (Merck, 1.06688.0100)] (20 min, 4°C), using either manufacturer-recommended or previously titrated antibody dilutions. Cells were then rinsed twice in cold FACS buffer. Flow cytometry was performed using LSRII (BD Biosciences), and data were analyzed using the FACS Diva program (BD Biosciences).

### 2D Co-Culture and Impedance Assay

HepG2-preS1-GFP target cells, monocytes, and HBV-specific TCR T cells were co-cultured for 24 h in AIM-V, 2% human AB serum supplemented with 100 IU/mL of rhIL-2. At 24 h, cells were collected and stained for the expression of different markers. To quantify HBV-specific TCR T cell killing by impedance measurements, HepG2.2.15 cells from a hepatoma cell line sustaining the full replication cycle of HBV were used in place of HepG2-preS1-GFP in cell culture medium without rhIL-2 supplementation. HepG2.2.15 cells were grown with or without monocytes for 24 h before non-specific T cells (Tns), Tdx, or EP HBV-specific TCR T cells were added to the culture. Impedance measurements were taken in real time for at least 24 h of co-culture with the respective T cells using xCELLigence RTCA DP (ACEA Biosciences, 00380601050). 2D and 3D co-cultures were performed in parallel. The cytotoxic activity of Tdx or EP HBV-specific TCR T cells was derived from the differential area under the curves (AUCs) of the HBV-specific TCR T cells with respect to Tns after obtaining the normalized cell index (NCI). NCI is calculated by taking the target growth index over the 24 h co-culture period and normalizing this to the time when T cells were first added to the culture. The presence of Tns takes into account the baseline non-specific killing.

### Formation of Tumor Cell Aggregates

HepG2-preS1-GFP cells were used to form cell aggregates following a previously reported protocol (50). Briefly, 1 × 10^6^ HepG2-preS1-GFP cells were added in a dropwise fashion into a 60-mm polystyrene Petri dish (Dow Corning, 430589) that was previously laser-ablated (VLS2.30 Desktop Laser, Universal Laser Systems) with a 100 × 100 microwell array spaced 0.5 mm by 0.5 mm apart (150 µm width, 150 µm depth). Prior to the addition of HepG2-preS1-GFP cells, the microwell dishes were cleaned with 70% ethanol to remove bubbles and free polymers that resided in the dishes, sterilized by UV-laser, and then treated with a 0.2% pluronic solution (Pluronic F108; Sigma-Aldrich, 542342) in PBS for 1 h to prevent cell attachment to the substrate. HepG2-preS1-GFP cells were cultured in 5 mL of R10 at 37°C and 5% CO_2_. After 3 days, cell aggregates were retrieved from the microwell dish and sieved through two cell strainers to yield aggregates with 40–100 µm diameters (51, 52).

### Fabrication of Microfluidic Device

Polydimethylsiloxane (PDMS) (Sylgard 184 silicone elastomer kit, Dow Corning) devices were fabricated using standard soft lithography methods that are similar to those previously described (53). Briefly, silicone elastomer and curing agent were mixed at a 10:1 ratio, degassed in a desiccator, poured into a 90-mm Petri dish that contained the silicon wafer mold (14), and cured overnight at 37°C. Devices were cut from the PDMS replica, and inlet/outlet ports were punched before sterilization *via* autoclave. After drying at 80°C overnight, the PDMS layers were plasma bonded (Covance Plasma System, Femto Science Inc.) to 24 mm × 50 mm glass cover slips (VWR, VWRI631-1574) and treated with 1 mg/mL of poly-d-lysine (Sigma-Aldrich, P7886) to enhance cell and collagen matrix binding to the microchannel walls and left at 80°C for at least 24 h to restore hydrophobicity. The final device consists of a middle gel channel flanked by two media channels. The gel channel is 1 mm wide, while the two fluidic channels are 500 µm wide. The three channels are interconnected, with a length of 14 mm and height of 120 µm (Figure 1A).

### 3D Microfluidic Co-Culture and Blocking Experiments

Monocytes were injected together with HepG2-preS1-GFP cell aggregates in a 2.5 mg/mL type I, rat tail collagen gel (354236, Corning) solution in the central hydrogel region of the microfluidic device (Figure 1A) (54). Approximately 5–10 cell aggregates were seeded in each device, together with monocytes at the concentration of 4 × 10^6^ cells/mL. After the injection of the hydrogel in the middle channel, the devices were kept in a humidity box at 37°C for 40 min to allow for gel polymerization by thermal cross-linking. R10 medium was then added into both lateral fluidic channels, and devices were allowed to stabilize overnight at 37°C and 5% CO_2_. Thereafter, T cells, suspended in AIM-V supplemented with 2% human AB serum and 100 IU/mL of rhIL-2, were added into one fluidic channel creating a pressure gradient that allowed HBV-specific TCR T cells to reach the interface of the hydrogel containing the target cells (Figure 1A). All experiments were performed in static (no-flow) culture conditions, and cell culture medium was replaced every day.

The following experimental configurations were first considered: (i) only HepG2-preS1-GFP (*Hep*), (ii) HepG2-preS1-GFP and monocytes (*Hep Mo*), (iii) HepG2-preS1-GFP and Ts (*Hep Ts*), (iv) HepG2-preS1-GFP and Tc (*Hep Tc*), (v) HepG2-preS1-GFP, Ts, and monocytes (*Hep Ts Mo*), and (vi) HepG2-preS1-GFP, Tc, and monocytes (*Hep Tc Mo*). HepG2-preS1-GFP death was then quantified as described in Section “Cell Staining, Confocal Imaging, and 3D Data Analysis” for the experiments of the different donors.

For blocking experiments, the same 3D assay was performed, but cell culture medium was supplemented with 10 µg/mL of anti-PD-L1-blocking (Biolegend, 329701) or anti-PD-1-blocking (eBioscience, 16-9989-82) antibodies or their respective isotype controls: Mouse IgG2b, κ isotype control (Biolegend, 400324), and Mouse IgG1, κ (eBioscience, 16-4714-82).

### Cell Staining, Confocal Imaging, and 3D Data Analysis

To detect and visualize cancer cell death in real time, the nuclear dye DRAQ7 (Biolegend, 424001) was added in the culture medium. HBV-specific TCR T cells were labeled with Cell Tracker Violet BMQC (Life Technologies, C10094) as previously described (14). The three cell types within the collagen compartment were imaged by confocal microscopy using a 20× magnification (LSM 780, Carl Zeiss), acquiring 3D image z-stacks of the tumor aggregate before the HBV-specific TCR T cells were introduced into the device (0 h), and then at 24 h after the addition of HBV-specific TCR T cells.

All confocal images where analyzed using Imaris 8.1 software (Bitplane). Target cell death was quantified by the proportion of dead nuclei in the GFP-labeled tumor aggregate as previously described (Figure 1B) (14). For each condition, the analysis of HepG2-preS1-GFP death was performed for at least three devices and plotted as the mean percentage of the dead target volume of the experiments for monocytes of three donors. The same data are also presented in terms of a “Dead Target Index,” where the percentage of dead target volume is normalized to the percentage of TCR^+^ T cells.

### Statistical Analysis

All data were calculated considering at least three ROIs (three to five target cell aggregates) for each device. Unless otherwise stated, the data were plotted as either the mean ± SEM or as box plots that show the 25th, 50th, and 75th percentile, as well as the minimum and maximum values using Prism (GraphPad software). Statistical analysis was determined using a two-tailed Student’s *t*-test and where appropriate, one-way ANOVA with Holm-Sidak’s multiple comparisons test. Only *P* values and adjusted *P* values (ANOVA) of less than 0.05 were taken as evidence of a statistically significant difference.

## Results

### PD-L1/PD-1 Expression on Monocytes, HepG2-preS1-GFP, and TCR T Cells

The PD-L1/PD-1 axis is an important immune checkpoint in HBV-HCC (18–20, 55–57). To determine if this axis is playing a role in our model, we first co-cultured Tdx and EP TCR T cells, HepG2-preS1-GFP and monocytes in 2D wells for 24 h as described in Section “Materials and Methods” and measured the surface expression of PD-L1 and PD-1 on the various cell types by flow cytometry.

**Figure 2:**
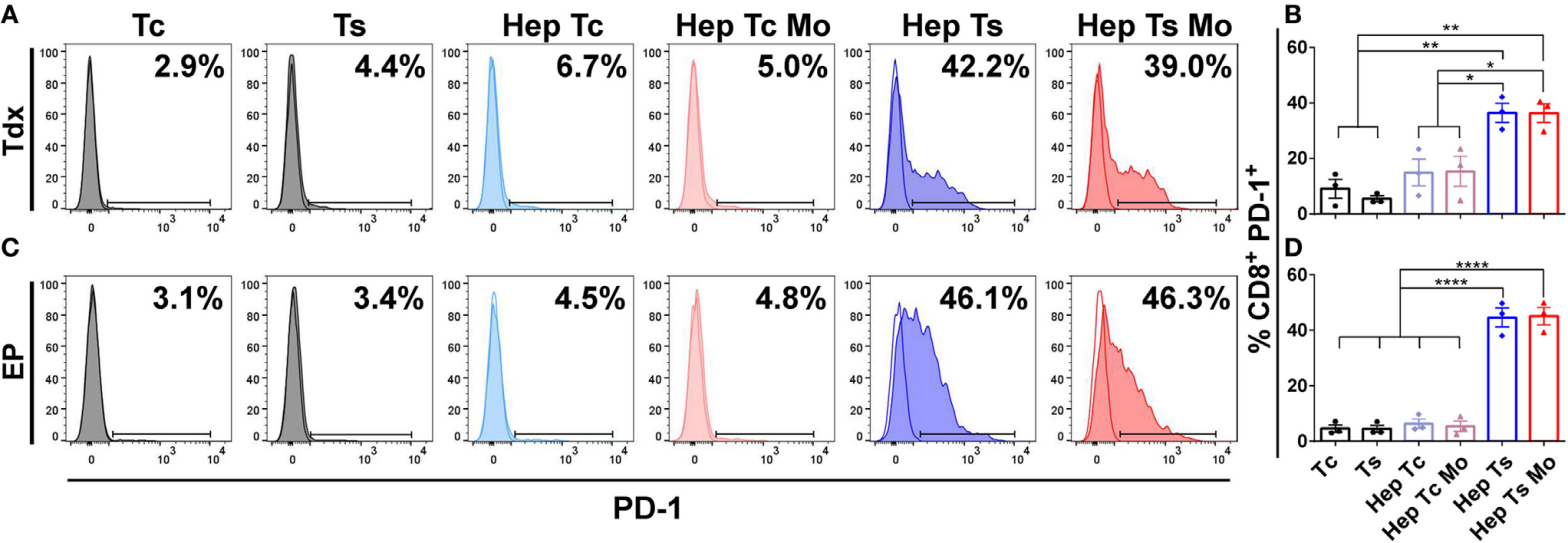
**(A,C)** Representative histograms of the flow cytometry data of CD8^+^ PD-1^+^ Tdx **(A)** and EP **(C)** HBV-specific TCR T cells at 24 h are shown. Tinted and non-tinted histograms, respectively, represent the stained sample or matched isotype control, where a horizontal line is drawn based on the isotype control to demarcate for PD-1^+^ cells. Percentage values of CD8^+^ PD-1^+^ cells are indicated. **(B,D)** Bar plots show the mean ± SEM of CD8^+^ PD-1^+^ TCR T cells of three donors for Tdx **(B)** or EP **(D)** HBV-specific TCR T cells; bars are drawn where a comparison was made with *P ≤ 0.05, **P ≤ 0.01 and ****P ≤ 0.0001. Statistical significance was evaluated by a one-way ANOVA with Holm-Sidak’s multiple comparisons test. EP, mRNA-electroporated; HBV, hepatitis B virus; Hep, HepG2-preS1-GFP; Mo, monocyte; Tc, control T cell; TCR T cells, T cell receptor-redirected T cells; Tdx, transduced; Ts, HBV-specific T cell.

At baseline, both Tdx and EP CD8^+^ HBV-specific TCR T cells (Ts) expressed negligible levels of PD-1 (Figure 2). However, upon co-culture with only target HepG2-preS1-GFP cells (*Hep Ts*) for 24 h, 36.6 ± 3.4% of Tdx Ts cells (Figures 2A,B) and 44.6 ± 3.4% of EP Ts cells (Figures 2C,D) were observed to upregulate PD-1 expression. Interestingly, the proportion of PD-1^+^ Tdx and EP Ts did not change significantly when monocytes were also in the co-culture (*Hep Ts Mo*), suggesting that PD-1 upregulation is independent of the presence of monocytes. Neither Tdx nor EP control HBV-specific TCR T cells (Tc) displayed a similar upregulation of PD-1 when in co-culture with either only target cells (*Hep Tc*) or target cells and monocytes (*Hep Tc Mo*). These data thus show that PD-1 upregulation on Tdx and EP Ts cells is due to TCR T cell activation through the specific engagement of the TCR with the target HepG2-preS1-GFP cells. More importantly, the data suggest that both types of TCR T cells could exhibit similar susceptibility to PD-L1/PD-1-based inhibition since comparable proportions of PD-1^+^ HBV-specific TCR T cells were observed.

**Figure 3:**
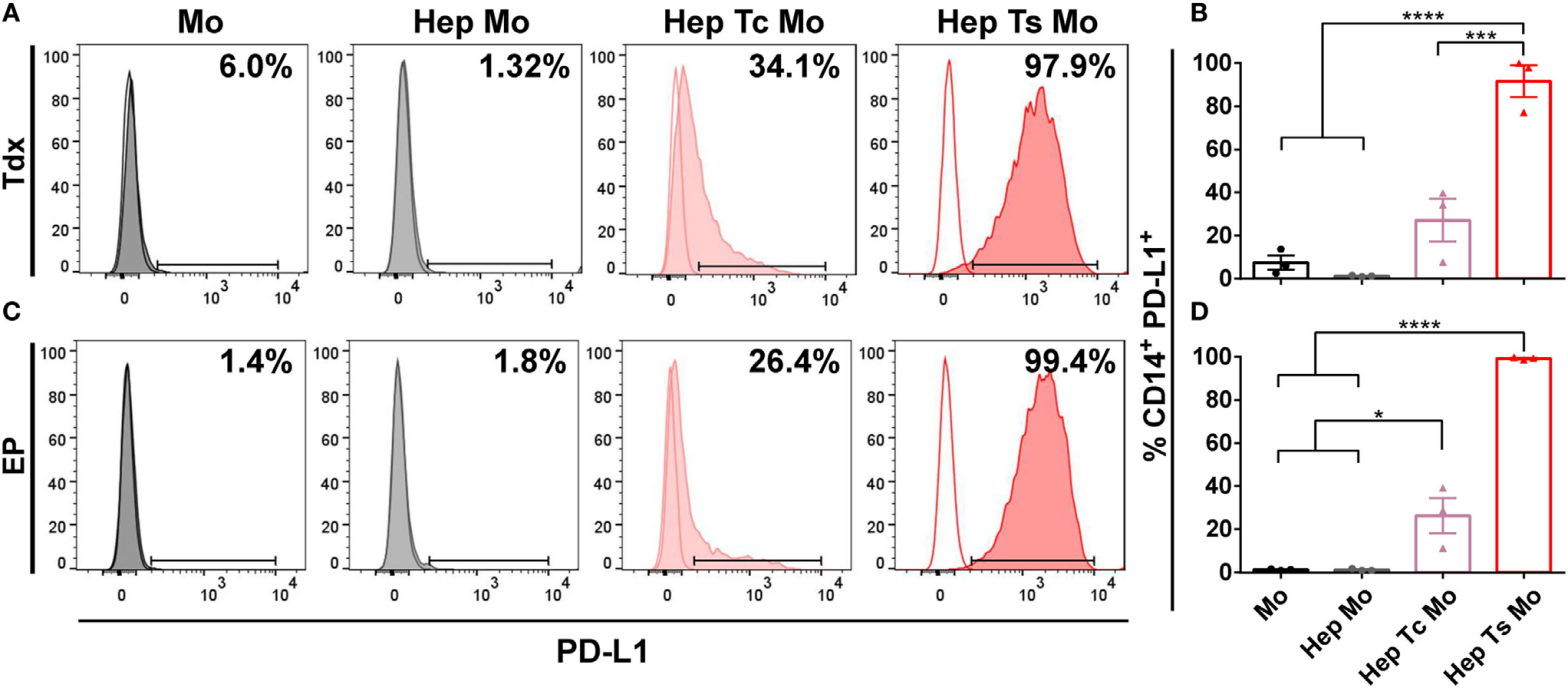
**(A,C)** Representative histograms of the flow cytometry data of CD14^+^ PD-L1^+^ monocytes for co-cultures with either Tdx **(A)** or EP **(C)** HBV-specific TCR T cells at 24 h are shown. Tinted and non-tinted histograms, respectively, represent the stained sample or matched isotype control, where a horizontal line is drawn based on the isotype control to demarcate for PD-L1^+^ cells. Percentage values of CD14^+^ PD-L1^+^ cells are indicated. **(B,D)** Bar plots show the mean ± SEM of PD-L1^+^ monocytes for co-cultures involving Tdx **(B)** or EP **(D)** HBV-specific TCR T cells; bars are drawn where a comparison was made with *P ≤ 0.05, ***P ≤ 0.001, and ****P ≤ 0.0001. Statistical significance was evaluated by a one-way ANOVA with Holm-Sidak’s multiple comparisons test. EP, mRNA-electroporated; HBV, hepatitis B virus; Hep, HepG2-preS1-GFP; Mo, monocyte; Tc, control T cell; TCR T cells, T cell receptor-redirected T cells; Tdx, transduced; Ts, HBV-specific T cell.

Corresponding with the increase in proportion of PD-1^+^ T cells, almost all monocytes upregulated PD-L1 expression when in co-culture with target cells and Ts (*Hep Ts Mo)* for either Tdx (Figures 3A,B) or EP (Figures 3C,D) Ts (91.6 ± 7.3% and 99.3 ± 0.3%, respectively). However, a significantly lower proportion of PD-L1^+^ monocytes was observed when in co-culture with target cells and Tc (*Hep Tc Mo)* for either Tdx or EP Tc (27.1 ± 9.9% and 26.2 ± 8.1%, respectively). Finally, monocytes cultured either alone (*Mo*) or with only target cells (*Hep Mo*) did not show any PD-L1 upregulation. These results thus suggest that like PD-1 upregulation, the increase in the proportion of PD-L1^+^ monocytes is related to their interaction with activated Ts that had contacted target cells. In addition, there was no significant change in PD-L1 expression on HepG2-preS1-GFP cells in the presence of Tdx or EP Ts with or without monocytes (Figure S1 in Supplementary Material), suggesting that monocytes would primarily mediate PD-L1-based effects.

### No Difference in Tdx and EP TCR T Cell Activity When Co-Cultured with Monocytes in 2D Cytotoxicity Assays

**Figure 4:**
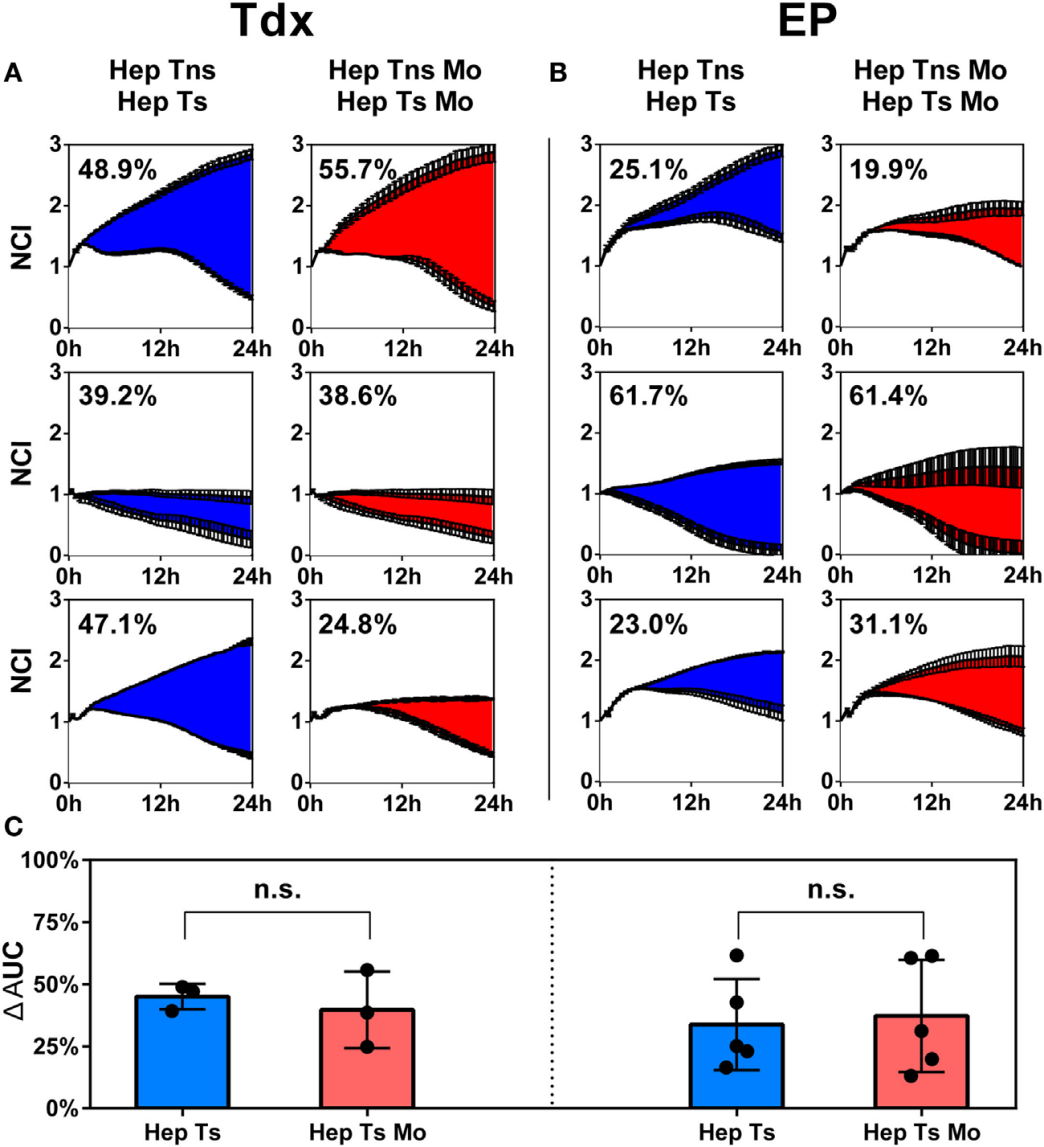
**(A,B)** Results from the impedance assay plotted as growth of the target cells over time for up to 24 h, measured as a NCI for Tns (top lines) and Tdx **(A)** or EP **(B)** HBV-specific TCR T cells (bottom lines). Each row represents a different donor. The numbers in each plot indicate the percentage difference of the AUC of target cell killing in HepG2-T cells co-cultures either in the absence (blue) or presence (red) of monocytes. Every curve is the average of at least two measurements for each condition and at least three experiments were performed. **(C)** Bar plots representing the average of all differential AUC measured in the experiments for Tdx (left panel) and EP (right panel) HBV-specific TCR T cells. Statistical significance was evaluated by a two-tailed *t*-test, with *P* < 0.05 taken as evidence of statistical significance. AUC, area under the curve; EP, mRNA-electroporated; HBV, hepatitis B virus; Hep, HepG2.2.15; Mo, monocyte; NCI, normalized cell index; n.s., not significant; TCR T cells, T cell receptor-redirected T cells; Tdx, transduced; Tns, non-specific T cells; Ts, HBV-specific T cell.

2D cytotoxicity assays to evaluate the effect of monocytes on HBV-specific TCR T cells were performed using an impedance killing assay as described in Section “Materials and Methods” (Figure S2 in Supplementary Material). Prior to culture, Tdx or EP TCR T cells were analyzed for both the proportion of CD8^+^ cells and the TCR expression using specific pentameric complexes for Ts or dextrameric complexes for Tc by flow cytometry. Gating on total lymphocytes, Tdx Ts were 84 ± 10% CD8^+^ and 39 ± 17% TCR^+^, while Tc were 74 ± 5% CD8^+^ and 37 ± 22% TCR^+^. As for EP T cells, Ts were 78 ± 5% CD8^+^ and 72 ± 5% TCR^+^, while Tc were 75 ± 7% CD8^+^ and 58 ± 10% TCR^+^ (Figure S3 in Supplementary Material). Results shown of the 2D cytotoxicity assays are the percentage reduction of the differential AUCs for the samples (*Hep Ts* or *Hep Ts Mo*) compared to the respective controls (*Hep Tns* or *Hep Tns Mo*) over the 24 h of co-culture (Figures 4A,B). When Tdx Ts cells were present, there was no statistically significant difference in the reduction of AUC in the absence (*Hep Ts*) or presence (*Hep Ts Mo*) of monocytes (45 ± 5.1% and 39.7 ± 15.5%, respectively) (Figure 4C; left panel). Similarly, no difference was observed when EP Ts cells were added to both the cultures of *Hep Ts* and *Hep Ts Mo* (33.8 ± 18.3% and 37.3 ± 22.6%, respectively) (Figure 4C; right panel). These data indicate that the addition of monocytes did not affect the cytotoxic activity of Ts in a 2D assay despite the induced expression of PD-1 on TCR T cells and PD-L1 on monocytes.

Since standard 2D assays have been reported to not fully recapitulate the cellular interactions that take place in complex systems, we therefore utilized a 3D microfluidic tumor model to evaluate the effect of monocytes on the functional activity of both Tdx and EP HBV-specific TCR T cells.

### Generation of 3D Microfluidic TME

The *in vitro* 3D tumor model was created by increasing the cellular diversity of the microfluidic system previously developed by Pavesi et al. through the incorporation of human primary monocytes (14). The present tumor model specifically involves a 3D co-culture of target HepG2-preS1-GFP cell aggregates, HBV-specific TCR T cells, and monocytes within a microfluidic device (Figure 1A).

**Figure 5:**
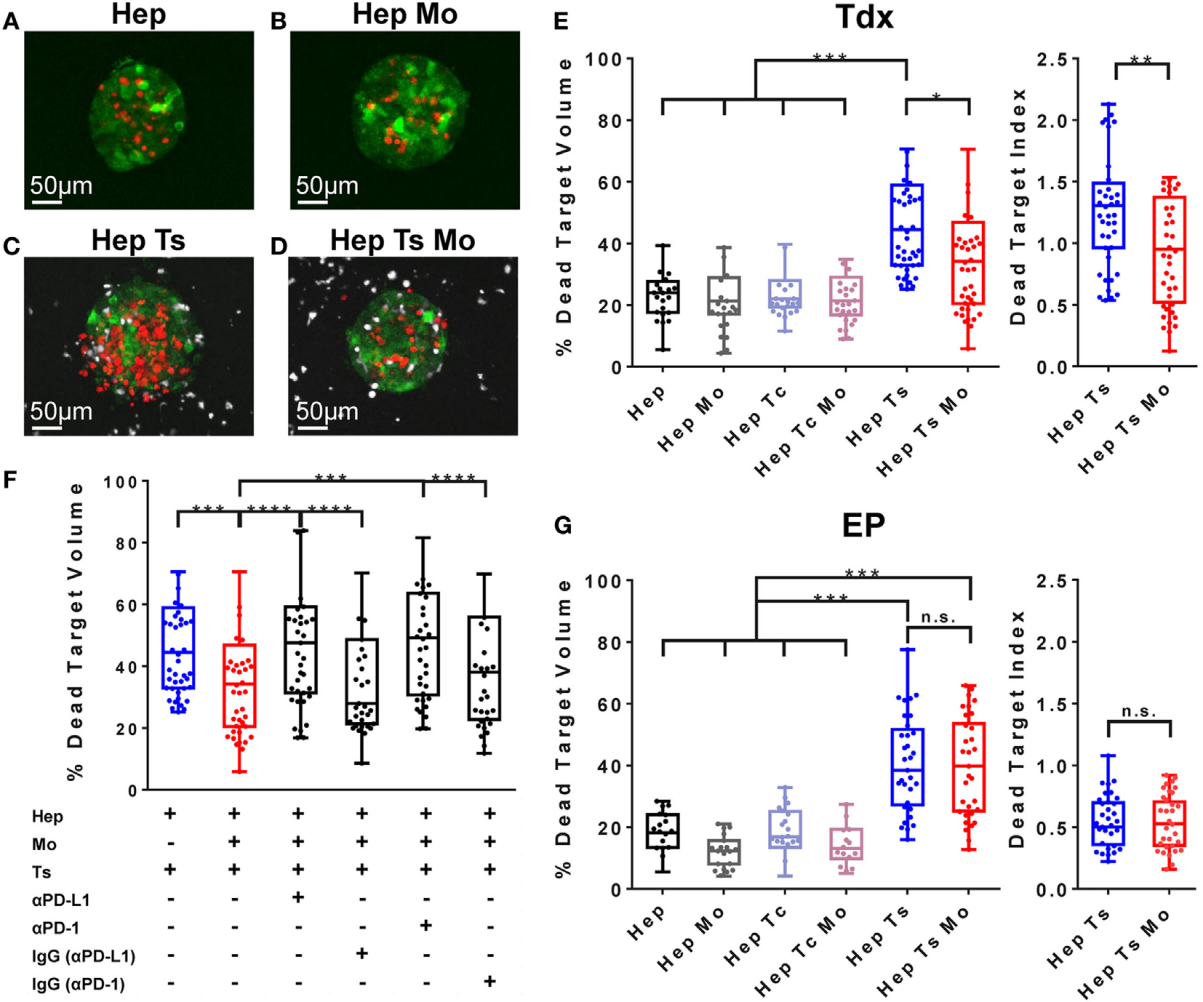
**(A–D)** Representative target cell aggregates of the conditions *Hep* **(A)**, *Hep Mo* **(B)**, *Hep Ts* **(C)**, and *Hep Ts Mo* **(D)**, in which the presence of dead target cells is DRAQ7^+^ (in red), HBV-specific TCR T cells are labeled with Cell tracker violet dye (in white), while monocytes are unlabeled. **(E)** Box plot of the percentage of dead target (HepG2-Pres1-GFP) volume after 24 h of co-culture with Tdx HBV-specific TCR T cells. Data in terms of a “Dead Target Index” are also shown where the percentage of dead target volume is normalized to the percentage of TCR^+^ T cells. **(F)** Box plot of the percentage of dead target volume after 24 h of treatment with Tdx HBV-specific TCR T cells with or without PD-L1- or PD-1-blocking antibody or their respective isotype (IgG) controls. **(G)** Box plot of the percentage of dead target (HepG2-Pres1-GFP) volume after 24 h of co-culture with EP HBV-specific TCR T cells. Data in terms of a “Dead Target Index” are also shown. Data points reflect the measured values of individual target cell aggregates and collectively represent the pooled results of three donors, where the 25th, 50th, and 75th percentiles as well as minimum and maximum values are indicated. Bars are drawn where a comparison was made with *P ≤ 0.05, **P ≤ 0.01, ***P ≤ 0.001, and ****P ≤ 0.0001. Statistical significance was evaluated by a one-way ANOVA with Holm-Sidak’s multiple comparisons test. EP, mRNA-electroporated; HBV, hepatitis B virus; Hep, HepG2-preS1-GFP; Mo, monocyte; n.s., not significant; Tc, control T cell; TCR T cells, T cell receptor-redirected T cells; Tdx, transduced; Ts, HBV-specific T cell.

Monocytes were suspended together with target cell aggregates in collagen gel, introduced into the central hydrogel region of the microfluidic device and cultured overnight. The following day, HBV-specific TCR T cells (Ts or Tc) were added into one of the fluidic channels and allowed to migrate toward the middle hydrogel region where the target cell aggregates and monocytes reside. The final cellular arrangement in the microfluidic platform mimics some features of the *in vivo* TME and allows for the observation and analysis of cell–cell interactions. DRAQ7 nuclear dye staining for dead cells revealed a good baseline viability of both monocytes and target cell aggregates with or without monocytes in the absence of HBV-specific TCR T cells (Figures 5A,B,E).

### Inhibitory Capabilities of Monocytes on Tdx TCR T Cell Cytotoxic Activity

Pavesi et al. demonstrated the specific lysis of HBV-HCC cells by TCR T cells (14). Consistent with these previous results, we observed 46.7 ± 8.9% of dead target volume for the *Hep Ts* configuration (Figures 5C,E), which is twofold higher than the 23.3 ± 4.1% of dead target volume for the target cell aggregates alone (*Hep*) (Figure 5E). No such increase in dead target volume was observed when target cell aggregates were co-cultured with monocytes (*Hep Mo*) or monocytes and Tc (*Hep Tc Mo*) (21.4 ± 5% and 22.4 ± 4.5%, respectively) (Figure 5E). This suggests that it is the engagement of the target cell specifically by Tdx Ts and not monocytes nor Tdx Tc, which resulted in target cell death. Interestingly, when monocytes were present (*Hep Ts Mo*), Tdx Ts killing activity was significantly inhibited (Figures 5D,E). Taken together, these results demonstrate that the 3D microfluidic model is a functional platform that can be used to screen and compare across different co-culture conditions to elucidate the role of monocytes in TCR T cell immunotherapy, whereas standard 2D cytotoxicity assays had failed to do so in this case.

### Blockade of PD-L1/PD-1 Axis Affects Monocyte and Tdx TCR T Cell Interaction

Following the observation that monocytes affect the interaction of HBV-specific Tdx Ts with target cell aggregates to result in reduced target cell lysis, a subsequent set of experiments was performed to address if monocytes were inhibiting Tdx Ts function through the PD-L1/PD-1 axis. Blocking antibodies against either PD-L1 or PD-1 were added in culture, and the death of target cell aggregates was measured as previously described (14). Blockade (Figure 5F) of either PD-L1 or PD-1 was observed to restore the killing capabilities of Tdx Ts (presenting a dead target volume of 46.8 ± 11% and 48.6 ± 9.8%, respectively), whereas addition of the matched isotype control antibodies had no effect on HBV-specific TCR T cell cytotoxic activity (presenting a dead target volume of 37.2 ± 11% and 37.0 ± 11.2%, respectively) (Figure S4 in Supplementary Material for representative confocal images of target cell aggregates). Therefore, these results suggest that monocytes affect Tdx Ts functionality through a PD-L1/PD-1-dependent mechanism.

### Monocytes Do Not Affect EP TCR T Cell Cytotoxic Activity

To verify if HBV-specific TCR T cell inhibition is a phenomenon specific for Tdx Ts and to investigate whether the same inhibition occurs in the case of EP Ts, a set of experiments was performed to compare between the two types of TCR T cell populations. As observed for Tdx HBV-specific TCR T cells, a twofold increase in cytotoxic activity was observed in the *Hep Ts* configuration (presenting a dead target volume of 38.8 ± 3.3%) with respect to the target cell aggregates alone (*Hep*) (presenting a dead target volume of 17.8 ± 2.9%). The co-culture with monocytes (*Hep Mo)* and/or Tc (*Hep Tc* or *Hep Tc Mo*) did not exhibit any increase in dead target volume over target cell aggregates alone, suggesting again that the cause of target cell death is the engagement of the target cell by EP Ts and not monocytes nor EP Tc. In contrast to the Tdx Ts co-culture, the presence of monocytes did not inhibit EP Ts in their killing activity (Figure 5G). Therefore, the data contribute supporting evidence toward the concept that functional differences do exist among differently produced TCR T cells that, in addition, might only be observed in a 3D setting.

## Discussion

Adoptive T cell immunotherapy has recently acquired clinical interest due to current positive outcomes for the treatment of liquid tumors (58–62). T cells isolated from patients may be engineered to express a specific TCR to target cancer cells and cause their lysis. However, different mechanisms inhibit the interaction of T cells with cancer cells, especially in the case of solid tumors. Among such obstacles, the TME and its cellular components appear to play the most crucial role (15, 29). In particular, monocytes are recruited from the circulation to the tumor site and are known to express PD-L1 that, when bound to PD-1 expressed on physiological T cells, may suppress T cell proliferation and cytotoxic activity toward cancer cells (19, 28, 29). Nevertheless, it remains to be clarified if such immunosuppression occurs with engineered TCR T cells. Previously, Pavesi et al. showed the establishment of a 3D microfluidic HBV-HCC tumor model to measure the cytotoxic ability of HBV-specific TCR T cells (14). In this article, we increased the complexity of the previous model by adding human primary monocytes to the HBV-HCC tumor model. We have thus developed a 3D *in vitro* TME that mimics the myeloid component of the *in vivo* intrahepatic TME, and for the first time, we demonstrate that 3D *in vitro* platforms can be used to help elucidate the role of monocytes in TCR T cell immunotherapy.

A pathological analysis of HBV-HCC patient-derived samples revealed that, compared with healthy tissue, there are significantly higher proportions of PD-L1^+^ monocytes in both intratumoral and peripheral regions (18–20, 56). Similarly in our work, there were higher proportions of PD-L1^+^ monocytes in the three-cell co-cultures of cancer cells, monocytes, and HBV-specific TCR T cells. While previous studies have suggested the inhibitory contribution of PD-L1 expressed on cancer cells (16, 18, 63), in our system, the proportion of PD-L1^+^ HepG2-preS1-GFP cells did not change significantly with the presence of HBV-specific TCR T cells and/or monocytes, and these proportions were much lower than those observed for monocytes. Therefore, our data suggest that it is the PD-L1 presented on monocytes that may have a primary impact on the cytotoxic activity of HBV-specific TCR T cells.

Our flow cytometry data on the PD-1 expression on HBV-specific TCR T cells show that PD-1 expression on HBV-specific TCR T cells is independent of the presence of monocytes and is likely to be regulated by signaling *via* the TCR (64, 65). Interestingly, although both Tdx and EP HBV-specific TCR T cells displayed the potential to be inhibited by monocytes, their ability to kill target cancer cells seemed unchanged regardless of the presence of monocytes in the 2D cytotoxicity assay.

Prompted by our null observations in a 2D system, we moved toward a 3D microfluidic tumor model, which, among several other advantages, is a physiological improvement from the 2D *in vitro* system (43, 45). We first established that in our hands we had set up a reliable system, observing that both Tdx and EP HBV-specific TCR T cells were able to effectively kill target HepG2-preS1-GFP cells, consistent with the previous report (14). We confirmed that the cytotoxic activity of TCR T cells is specifically associated with their recognition of the HBs183-191 peptide, and, more importantly, we found that when monocytes were added to the system, Tdx HBV-specific TCR T cells displayed decreased cytotoxic activity toward HepG2-preS1-GFP cells.

Moreover, in line with the PD-L1/PD-1 expression profiles observed, this Tdx HBV-specific TCR T cell inhibition was abrogated when PD-L1/PD-1 signaling was blocked with a blocking antibody against either PD-L1 or PD-1. Notably, Tdx HBV-specific TCR T cell cytotoxic activity was restored by the same degree with PD-L1 or PD-1 blocked, supporting the hypothesis that PD-L1 is the key ligand involved in the observed Tdx HBV-specific TCR T cell inhibition.

The predominant role of monocyte PD-L1 is reinforced when we compared between the (i) three-cell type co-culture with a blocking antibody against either PD-L1 or PD-1, and the condition of (ii) only Tdx HBV-specific TCR T cells and target cells. If PD-L1 on target cells had contributed toward Tdx HBV-specific TCR T cell inhibition, then the Tdx HBV-specific TCR T cell cytotoxic activity observed in the former (i) would have been higher than in the latter (ii). This, however, was not the case here where the difference between (i) and (ii) was not statistically significant. Therefore, data strongly suggest that the PD-L1 expressed on HepG2-preS1-GFP cells did not fundamentally contribute to Tdx HBV-specific TCR T cell inhibition.

Intriguingly, different from Tdx HBV-specific TCR T cells, monocytes did not suppress the cytotoxic activity of EP HBV-specific TCR T cells in our microfluidic tumor model. These findings are of clinical interest, bearing in mind the ease of generating EP TCR T cells and their inherent self-limiting cytotoxicity due to their transient TCR expression (12). Moreover, the differential effect of monocytes on the two TCR T cell types likely stems from a difference in their activation status resulting from the use of different engineering methodologies and processes of T cell expansion and culture (11, 12). In particular, Tdx HBV-specific TCR T cells were expanded for at least 2 weeks with a γ-chain (γc) cytokine cocktail comprising IL-2, IL-7, and IL-15 that may have caused the expansion of cells that are more sensitive toward PD-1 inhibition. In contrast, EP HBV-specific TCR T cells were cultured with only IL-2 and for only up to a week. A similar phenomenon was observed by Kinter et al. where T cells that were treated with a similar cytokine cocktail as our Tdx T cells upregulated PD-1 that upon ligation displayed inhibited T cell effector functions compared to untreated T cells (66). Further studies that explore differential expression of exhaustion markers could help elucidate the diverse behavior of differently produced TCR T cells that we observed.

Our findings confirm the clear advantage of having a more realistic 3D tumor model over classical 2D platforms to screen for different therapeutic approaches as shown here for differently produced TCR T cells. In fact, similar limitations of the 2D assays were also discussed in the study by Pavesi et al. where the 2D cytotoxicity assay showed an overestimation of T cell killing and an inability to measure the effect of different oxygen levels on T cell behavior (14). Consistent observations regarding the sensitivity of 2D versus 3D cultures were also made in the context of chemotherapeutic agents, where the effects of drugs observed in 3D were dramatically decreased compared to the effects shown by the same drugs in a 2D setting (67–70).

Indeed, compared to a 2D set-up, the 3D microfluidic *in vitro* immunogenic TME we developed more closely mimics the physiological *in vivo* setting as the HBV-HCC cells are presented as aggregates in a 3D matrix and potentially allows for one to investigate the influence of different TME-based features or cellular players on cancer therapies. With this spatial organization, cells at the outer rim are the first to be targeted and lysed by HBV-specific TCR T cells, while those at the core are less susceptible to contact-dependent lysis. In contrast, the standard 2D set-up consists of HBV-HCC cells grown as a monolayer that is completely overlaid by the HBV-specific TCR T cells, thus allowing for faster and more successful killing as TCR T cells settle directly onto HBV-HCC cells by gravity without the resistance they would otherwise face when migrating in 3D or entering a 3D cell aggregate. Furthermore, in comparison to conventional 3D tumor cultures, our 3D microfluidic system permits the increase of architectural complexity of the TME by adding more cellular players (monocytes in this case) and allows one to investigate the influence of each player on cancer therapies.

Future experiments can be performed to clarify the mechanisms involved in the interactions of monocytes with both TCR T cell types. This would allow us to use our observations to optimize the currently described therapeutic strategies and also extend these studies more generally to other cancers. However, our results already have important implications for clinical therapies as demonstrated by the capacity of our 3D microfluidic preclinical model to predict differences in the *in vivo* interaction of different TCR T cell types with monocytes, thus providing therapeutic value by identifying the most efficacious form of TCR T cell immune therapy for personalized treatment.

## Ethics Statement

This study was carried out in accordance with the recommendations of National University of Singapore Institutional Review board with written informed consent from all subjects. All subjects gave written informed consent in accordance with the Declaration of Helsinki. The protocol was approved by the National University of Singapore Institutional Review board.

## Author Contributions

Conceptualization: SL, GA, AP, AT, AB, RK and SCW; methodology: SL, GA and EC; investigation: SL, GA, EC; formal analysis: SL and EC; writing—original draft: SL, GA and EC; writing—review and editing: SL, GA, EC, AP, AT, AB, RK and SCW; funding acquisition: AB, RK and SCW; resources: AB, RK and SCW; supervision: GA, AB, RK and SCW.

## Conflict of Interest Statement

The authors declare that the research was conducted in the absence of any commercial or financial relationships that could be construed as a potential conflict of interest.

## Abbreviations

TCR T cell, T cell receptor-redirected T cell; EP, electroporated; Tdx, transduced; TME, tumor microenvironment.
