# Corrigendum: Characterizing the Role of Monocytes in T Cell Cancer Immunotherapy Using a 3D Microfluidic Model

**DOI:** 10.3389/fimmu.2018.01719

**Published:** 2018-07-24

**Authors:** Sharon Wei Ling Lee, Giulia Adriani, Erica Ceccarello, Andrea Pavesi, Anthony Tanoto Tan, Antonio Bertoletti, Roger Dale Kamm, Siew Cheng Wong

**Affiliations:** ^1^BioSystems and Micromechanics IRG, Singapore-MIT Alliance for Research and Technology, Singapore, Singapore; ^2^Department of Microbiology and Immunology, Yong Loo Lin School of Medicine, National University of Singapore, Singapore, Singapore; ^3^Singapore Immunology Network (SIgN), Biomedical Sciences Institute, Agency for Science, Technology, and Research, Singapore, Singapore; ^4^Institute of Molecular and Cell Biology, Agency for Science, Technology, and Research, Singapore, Singapore; ^5^Programme of Emerging Infectious Diseases, Duke-NUS Medical School, Singapore, Singapore; ^6^Department of Biological Engineering, Massachusetts Institute of Technology, Cambridge, MA, United States

**Keywords:** microfluidics, monocytes, T cell receptor-redirected T cells, immunotherapy, immune checkpoint, PD-L1, tumor microenvironment

In our original research article, there was an error in the *Materials and Methods* section, subsection *3D Microfluidic Co-Culture and Blocking Experiments*, where it was written that 100 µg/mL of anti-PD-L1-blocking or anti-PD-1-blocking antibody or their respective isotype control was used. The correct concentration is 10 µg/mL. The authors apologize for this error and state that this does not change the scientific conclusions of the article in any way.

The original article has been updated.

## Conflict of Interest Statement

The authors declare that the research was conducted in the absence of any commercial or financial relationships that could be construed as a potential conflict of interest.

