# Supplementary material for: Characterizing the Role of Monocytes in T Cell Cancer Immunotherapy Using a 3D Microfluidic Model
Source: Front Immunol. 2018 Mar 6;9:416. doi: 10.3389/fimmu.2018.00416 (PMC5845585; doi:10.3389/fimmu.2018.00416)
Supplement: Supplementary file 1 [file presentation_1.PDF]

Supplementary Material

Characterizing the Role of Monocytes in T Cell Cancer Immunotherapy Using a 3D Microfluidic Model

Lee Sharon Wei Ling[1,2,3,†], Adriani Giulia[1,†], Ceccarello Erica[2,4,†], Pavesi Andrea[4], Tan Anthony Tanoto[5], Bertoletti Antonio[5], Kamm Roger Dale[1,6,*], Wong Siew Cheng[2,3,*]

[1]BioSystems and Micromechanics IRG, Singapore-MIT Alliance for Research and Technology, Singapore.

[2]Department of Microbiology and Immunology, Yong Loo Lin School of Medicine, National University of Singapore, Singapore.

[3]Singapore Immunology Network (SIgN), Biomedical Sciences Institute, Agency for Science, Technology, and Research, Singapore.

[4]Institute of Molecular and Cell Biology, Agency for Science, Technology, and Research, Singapore.

[5]Programme of Emerging Infectious Diseases, Duke-NUS Medical School, Singapore.

[6]Department of Biological Engineering, Massachusetts Institute of Technology, Cambridge, MA, United States.

[†]These authors contributed equally

***Correspondence:**

Wong Siew Cheng

wong_siew_cheng@immunol.a-star.edu.sg

Kamm Roger Dale

rdkamm@mit.edu

1 Supplementary Figures

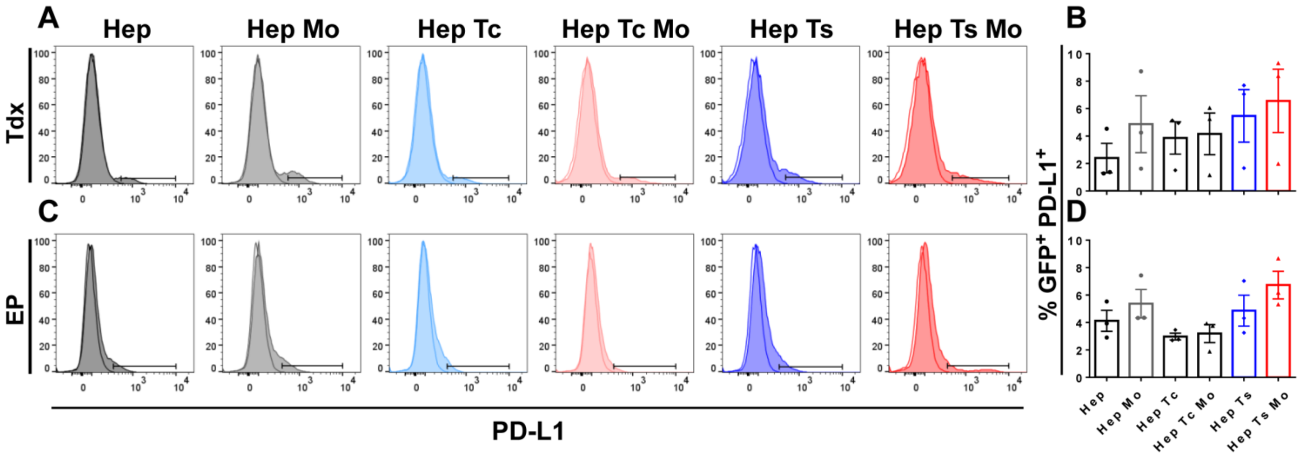

Supplementary Figure 1: Less important role of PD-L1 expression on HepG2-preS1-GFP cells in the interaction between monocytes and HBV-specific TCR T cells. (A, C) Representative histograms of the flow cytometry data of PD-L1$^+$ HepG2-preS1-GFP cells for co-cultures with either Tdx (A) or EP (C) HBV-specific TCR T cells at 24 h are shown. Tinted and non-tinted histograms respectively represent the stained sample or matched isotype control, where a horizontal line is drawn based on the isotype control to demarcate for PD-L1$^+$ cells. (B, D) Bar plots show the mean ± SEM of PD-L1$^+$ HepG2-preS1-GFP cells for co-cultures involving Tdx (B) or EP (D) HBV-specific TCR T cells. Statistical significance was evaluated by a one-way ANOVA with Holm-Sidak's multiple comparisons test, with $P \leq 0.05$ taken as evidence of statistical significance. EP, mRNA-electroporated; HBV, hepatitis B virus; Hep, HepG2-preS1-GFP; Mo, monocyte; Tc, control T cell; TCR T cells, T cell receptor-redirected T cells; Tdx, transduced; Ts, HBV-specific T cell.

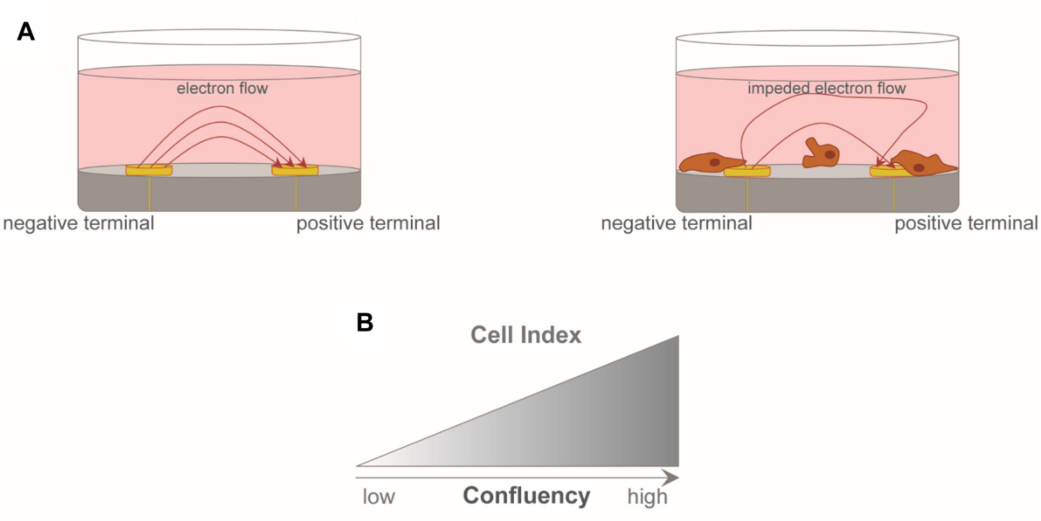

Supplementary Figure 2: Scheme of xCELLigence RTCA DP. (A) In the presence of culture medium, when a current is applied, electrons move freely from the negative to the positive terminal. When the HBV-producing hepatoma cell line is seeded, the electron flow is impeded, resulting in an increase in the Cell Index. (B) The Cell Index is proportional to the impedance measured in the well, indicating the confluency of the adhering cells. When HBV-specific TCR T cells are added to the culture, the HBV-producing target cells are lysed such that electron flow is restored and the Cell Index decreases. HBV, hepatitis B virus; TCR T cells, T cell receptor-redirected T cells.

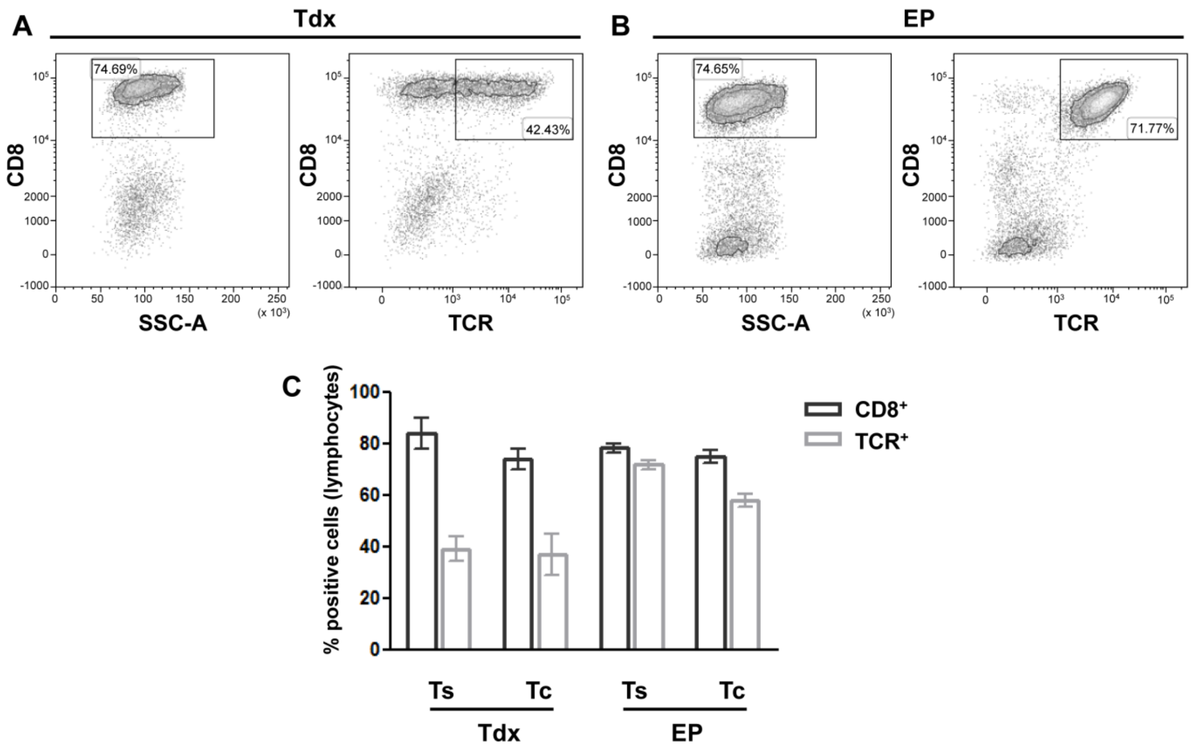

Supplementary Figure 3: Transduced (Tdx) and mRNA-electroporated (EP) HBV-specific TCR T cell expression of a HBV s183-191 or core18-27-specific TCR does not influence their PD-1 expression. Prior to every 2D or 3D assay, HBV-specific TCR T cells were tested for their CD8 and HBV-specific TCR expression using flow cytometry. Representative dot plots of CD8 and HBV s183-191-specific MHC pentameric staining for Tdx (A) and EP (B) HBV-specific TCR T cells (Ts) are shown. The gate for TCR positivity is set based on MHC pentameric staining of T cells that are not retrovirally transduced/mRNA-electroporated. Percentage values of $CD8^{+}$ T cells from total lymphocytes (left panels in A, B) and percentage of TCR^{+} T cells from total lymphocytes (right panels in A, B) are indicated. (C) Bar plots show the mean ± SEM of the percentage of $CD8^{+}$ HBV-specific TCR T cells and percentage of TCR^{+} T cells from total lymphocytes for all TCR T cells used in the experiments. HBV, hepatitis B virus; Hep, HepG2-preS1-GFP; Mo, monocyte; Tc, control T cell; TCR T cells, T cell receptor-redirected T cells; Ts, HBV-specific T cell.

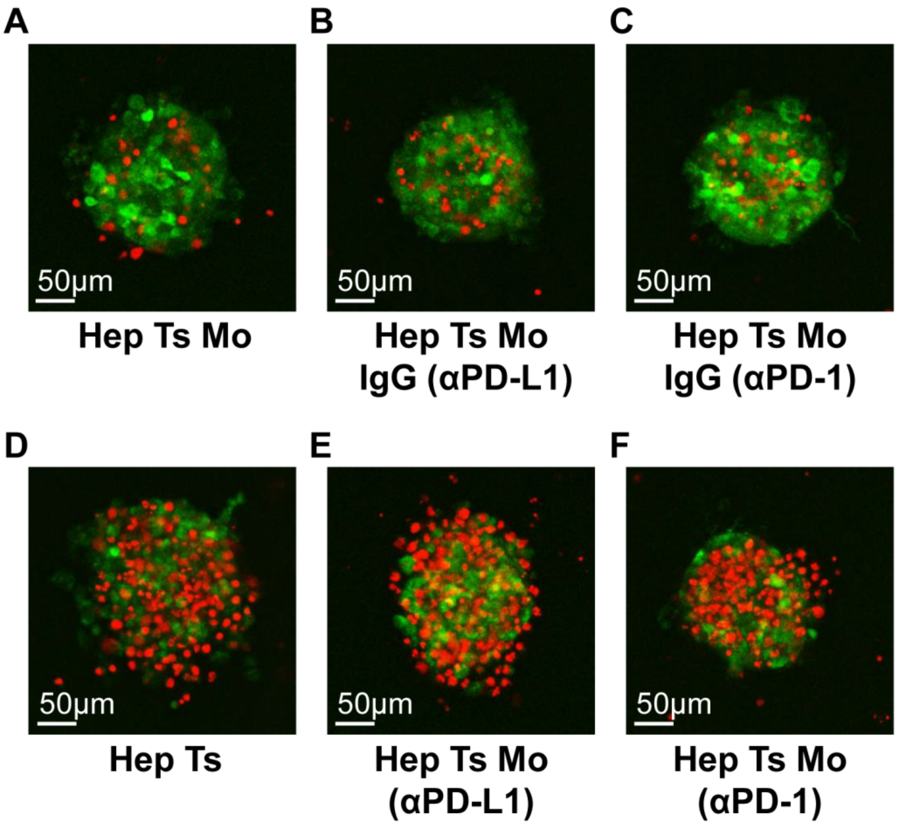

Supplementary Figure 4: Tdx HBV-specific TCR T cell cytotoxic activity. HBV-specific TCR T cell killing of target cell aggregates is indicated by the presence of GFP-dim DRAQ7^{+} (red) cells, where DRAQ7 is a nuclear dye that labels for dead cells. Images are representative aggregates of Tdx HBV-specific TCR T cell-associated cultures for the indicated conditions (A-F). Both HBV-specific TCR T cells and monocytes are not shown. HBV, hepatitis B virus; Hep, HepG2-preS1-GFP; Mo, monocyte; Tc, control T cell; TCR T cell, T cell receptor-redirected T cell; Tdx, transduced; Ts, HBV-specific T cell.
